# Stimulus valence, episodic memory, and the priming of brain activation profiles in borderline personality disorder

**DOI:** 10.1017/S0033291721001136

**Published:** 2021-04-16

**Authors:** Morgan Szczepaniak, Asadur Chowdury, Paul H. Soloff, Vaibhav A. Diwadkar

**Affiliations:** 1 Department of Psychiatry & Behavioral Neurosciences, Wayne State University, Detroit, USA; 2 Department of Psychiatry, University of Pittsburgh, Pittsburgh, USA

**Keywords:** Borderline Personality Disorder, Episodic Memory, Negative Valence, Positive Valence, Repetition Priming

## Abstract

**Background.:**

Borderline personality disorder (BPD) is characterized by instability in affective regulation that can result in a loss of cognitive control. Triggers may be neuronal responses to emotionally valenced context and/or stimuli. ‘Neuronal priming’ indexes the familiarity of stimuli, and may capture the obligatory effects of affective valence on the brain’s processing system, and how such valence mediates responses to the repeated presentation of stimuli. We investigated the effects of affective valence of stimuli on neuronal priming (i.e. changes in activation to repeated presentation of stimuli), and if these effects distinguished BPD patients from controls.

**Methods.:**

Forty BPD subjects and 25 control subjects (age range: 18–44) participated in an episodic memory task during fMRI. Stimuli were presented in alternating epochs of encoding (six images of positive, negative, and neutral valence) and recognition (six images for ‘old’ *v.* ‘new’ recognition). Analyses focused on inter-group differences in the *change in activation* to repeated stimuli (presented during Encoding and Recognition).

**Results.:**

Relative to controls, BPD showed greater priming (generally greater *decrease* from encoding to recognition) for *negatively* valenced stimuli. Conversely, BPD showed less priming for *positively* valenced stimuli (generally greater increase from encoding to recognition).

**Conclusion.:**

Plausibly, the relative familiarity of negative valence to patients with BPD exerts an influence on obligatory responses to repeated stimuli leading to repetition priming of neuronal profiles. The specific effects of valence on memory and/or attention, and consequently on priming can inform the understanding of mechanisms of altered salience for affective stimuli in BPD.

## Introduction

Borderline personality disorder (BPD) is characterized by instability in affective regulation and self-image (Lieb, Zanarini, Schmahl, Linehan, & Bohus, 2004). These psychological traits express themselves in abnormal functional responses and network interactions in the BPD brain (Sala et al., 2009). Task-based functional magnetic resonance imaging (fMRI) studies are particularly valuable in evoking altered response profiles in BPD, and relying on behavioral domains like episodic memory to evoke responses can be particularly useful (Yonelinas, Ranganath, Ekstrom, & Wiltgen, 2019). Episodic memory requires participants to encode and subsequently recognize previously presented stimuli, and can evoke implicit responses to stimulus valence. Stimulus repetition leads to adaptations in fMRI-measured responses (Grill-Spector & Malach, 2001; Krekelberg, Boynton, & van Wezel, 2006) that can be relatively invariant of task demands (Xu, Turk-Browne, & Chun, 2007). Thus, changes in fMRI-indexed responses provide viable mesoscopic representations of how neuronal responses attenuate to repeated exposure to stimuli (a process generally referred to as neuronal priming) (de Aquino Ferreira, Queiroz Pereira, Neri Benevides, & Aguiar Melo, 2018). Processes of adaptation (or enhancement) under stimulus repetition are a mechanism that in their sensitivity to stimulus valence, may reveal fundamental properties of cognitive/affective architectures in health and illness (Barron, Garvert, & Behrens, 2016). In the context of BPD, stimulus valence interferes with *effortful* task processing (Gvirts et al., 2015; Soloff, Abraham, Ramaseshan, Burgess, & Diwadkar, 2017b; Winter et al., 2015) but the modulatory effects of stimulus *valence* on priming may be a highly noteworthy area of inquiry.

### Neuronal priming

The repeated presentation of a stimulus leads to changes in behavioral or neuronal responses, with each subsequent presentation increasing stimulus familiarity and processing efficiency. This process is generally referred to as priming (Fiebach, Gruber, & Supp, 2005). Priming is thought to result from a ‘sharpening’ effect on neurons (an increase in the efficiency of processing) that encode specific (and repeated) stimuli in neuronal networks (Martens & Gruber, 2012). This sharpening results in faster response times and generally, reductions in direct or surrogate (e.g. fMRI) measures of neuronal activity. Familiarity exerts similar effects because neuronal representations of familiar stimuli can be more efficiently processed during subsequent recall/recognition. In experimental settings, (repetition) priming typically leads to an attenuation of activation from the first presentation to the second presentation (Garcia-Marques, Prada, & Mackie, 2016; Lebreton et al., 2012).

Priming and familiarity are likely to be mediated by affect or affective salience. For instance, healthy subjects exhibit a strong preference for benevolent experiences or positive affect (Garcia-Marques et al., 2016), resulting in positively valenced stimuli being tagged as more salient and therefore more familiar. Indeed, healthy subjects exhibit neuronal priming (attenuation of activation) in the presence of positive affect (Biss & Hasher, 2011), evidence in support of a relationship in the healthy brain between positive affect and familiarity. These effects relating to positive valence motivate the possibility of converse effects related to *negative* valence in the context of disorders of emotion dysregulation such as BPD. BPD patients (as with patients of other disorders of emotion dysregulation) are characterized by a ‘negative bias’, wherein negatively valenced stimuli are more salient (and presumably more ‘familiar’, see below) (Bortolla et al., 2020). This contraposition in the relevance of stimuli motivates the possibility that neuronal priming in BPD may be greater for negatively valenced stimuli (though this question has not been directly tested). In contrast to priming, repetition enhancement relates to an increase in neuronal responses to the subsequent presentation of stimuli. This phenomenon has been observed in the context of willful or spontaneous episodic memory retrieval, and in regions such as the parietal and posterior cingulate cortices (Weymar, Bradley, Sege, & Lang, 2018). Outside of tasks with episodic memory demands, enhancement of neuronal responses is observed in the *early* stages of the repetition of unfamiliar stimuli (e.g. words) (Lebreton et al., 2012; Martens & Gruber, 2012). This enhancement appears to be a pre-cursor in the process of familiarity, wherein an early sharpening of neuronal responses is needed before a subsequent level of familiarity (and presumably priming) is reached for priming to occur (Henson et al., 2003; Subramaniam, Faust, Beeman, & Mashal, 2012). It is plausible that just as priming is an index of implicit or obligatory familiarity, enhancement reflects implicit (short-term) novelty.

### Relevance for borderline personality disorder

BPD results from a combination of biological temperament, genetics, and psychosocial influences. Childhood trauma and sexual abuse are factors in its development suggesting that most patients are frequently exposed to *negative* life experiences (de Aquino Ferreira et al., 2018). Indeed, as noted negative affect and traumatic life experiences are hypothesized to be more ‘familiar’ to BPD patients (Bertsch, Hillmann, & Herpertz, 2018), a familiarity that certainly impacts activation to negative stimuli in specific experimental contexts (de Aquino Ferreira et al., 2018). Multiple studies confirm that negatively valenced stimuli are more salient in BPD, and exert a multitude of diverse effects in episodic memory, attention, and affective processing tasks (Silbersweig et al., 2007; Soloff et al., 2017a, 2017b).

Selected studies in BPD patients have used fMRI to quantify activation changes associated with the repeated presentation of negatively valenced stimuli. These studies have relied on *explicit emotional subjectivity* in an effort to understand the relationship between behavioral ‘habituation’, that is attenuation of the behavioral response to repeated stimuli, and changes in fMRI activation (Denny et al., 2018; Koenigsberg et al., 2014). Traditional studies of repetition priming rarely enforce the *explicit* evaluation of the attribute of interest. Rather, they rely on the *implicit* effects of such attributes under repetition (Poldrack & Gabrieli, 2001). Accordingly, the current investigation focused on assessing the implicit effects of un-evaluated/un-rated stimulus valence on priming effects assessed in the context of a traditional episodic *memory* task. Following the presentation of scenes during Encoding epochs, participants judged whether subsequently presented scenes in Recognition epochs were shown in the preceding Encoding epoch (i.e. ‘old’ or ‘new’). No other explicit evaluations were demanded from participants. By focusing on inter-group differences in priming as they related to the normed valence of the stimuli, it was possible for us to evaluate the modulatory effects of positive or negative valence on ‘neuronal’ priming in BPD. If priming in this context is driven by the ‘familiarity’ associated with positively valenced stimuli (in healthy controls) but with negatively valenced stimuli (in BPD), we would predict greater priming for positive valence in controls, but greater priming for negative valence in BPD. Thus, the nature of the task permits the assessment of the obligatory effects of valence on the priming of brain activation profiles in BPD.

## Methods

### Inclusion criteria

This study’s procedures were approved by both Wayne State University and the University of Pittsburgh Institutional Review Boards. Sixty-five subjects’ data were utilized from an ongoing longitudinal study of BPD subjects recruited through psychiatric outpatient clinics and advertisements in the surrounding community (Soloff, White, Omari, Ramaseshan, and Diwadkar, 2015). The study was comprised of 25 HC and 40 BPD subjects between the ages of 18 and 44. The HC group was made up of 23 females and two males, and the BPD group was made up of 34 females and six males (see online Supplementary Table S1). These numbers are nearly representative of the morbidity of BPD, as women comprise approximately 75% of all clinical BPD patients (American Psychiatric Association, 2013). All subjects gave written informed consent. BPD subjects were required to meet the criteria for a probable or definite lifetime diagnosis of BPD from the International Personality Disorders Examination (IPDE) (Loranger, 1994). In addition, they were required to have a definite current diagnosis of BPD from the Diagnostic Interview for Borderline Patients-Revised (DIB-R), using a 2-year timeframe (Zanarini, Gunderson, Frankenburg, & Chauncey, 1989). The Structured Clinical Interview for DSM-IV (SCID) was used to determine the co-morbidity of Axis I disorders (First, 1997). Healthy controls were free of current or lifetime Axis I or Axis II diagnoses, and were therefore free of psychoactive medications, although BPD subjects could remain on any current psychoactive medications. All subjects were required to test negative to abusive drugs through urine toxicology, and to pregnancy tests if applicable (Soloff et al., 2015).

### Exclusion criteria

Exclusion criteria included the following: (1) a lifetime (past or current) Axis I diagnosis of schizophrenia, delusional (paranoid) disorder, schizoaffective disorder, any bipolar disorder (I, II, mixed, manic, or depressed), or psychotic depression; (2) a current DSM-IV diagnosis of substance dependence or any current drug- or alcohol-related CNS deficits (a DSM-IV diagnosis of substance abuse was permitted so long as the subject had been abstinent for one week, showed no signs of withdrawal, and had a clean urine toxicology drug screen (MedTox) at the time of the scan); (3) clinical evidence of CNS pathology of any etiology, including acquired or developmental deficits or seizure disorder; (4) physical disorders or treatments with the known psychiatric consequence (e.g. hypothyroidism, steroid medications); (5) borderline mental retardation (IQ < 70 on the Wechsler Adult Intelligence Scale); (6) standard exclusion criteria for MRI scans include the following: ferromagnetic implants such as cardiac pacers, cochlear implants, aneurysm clips, history of metal in eyes or other ferromagnetic body artifacts; inability to fit in the scanner due to obesity, claustrophobia or inability to tolerate brief confinement in the scanner; inability to co-operate with instructions (Soloff et al., 2015).

### Imaging specifications

All anatomical images were acquired on a 3.0 T Siemens Trio system in the axial plane parallel to the AC-PC line using a 3D MPRAGE sequence (TE/TI/TR = 3.29 m/900 m/2200 m, flip angle = 9, isotropic 1 mm^3^ voxel, 192 axial slices, matrix size = 256 × 192). The fMRI data for this experiment were acquired in the axial plane using gradient-echo EPI (TR = 2000 m, TE = 30 m, flip angle = 70 deg, 30 slices, slice thickness = 3.1 mm, 3 mm × 3 mm in-plane, matrix size = 64 × 64) (Soloff et al., 2015).

### fMRI paradigm procedure

An episodic memory task with a typical experimental design was used to assess fMRI activation profiles associated with the ENCoding and RECognition of episodic memories for International Affective Pictures System (IAPS) pictures (Lang, Bradley, & Cuthbert, 2008; Soloff et al., 2015). A total of 81 pictures were used in the entire experiment, 27 each were classified as negative, neutral, or positive valence. The images drawn for the polar valences (negative and positive) that were the focus of this inquiry differed in their normative ratings of valence (1.95 *v*. 7.37) but were chosen to be equivalent in terms of their rated arousal (6.87 *v*. 6.13). During the paradigm, 54 pictures were presented once during each of nine encoding epochs. Twenty-seven were reused in the subsequent recognition epochs as targets, and an additional 27 pictures were used as foils. During each encoding epoch, six pictures (two from each of Negative and Positive valence, and two Neutral images used as fillers) were presented for 4 s with a 500–1500 ms randomly jittered inter-stimulus interval, for a total of 27 s per epoch. A brief retention interval (8 s) preceded the recognition epoch, in which three of the pictures were targets (one of each valence class) and three were foils (one of each valence class). Participants were asked to indicate if the pictures were presented in the encoding epoch immediately preceding it (‘old’ for targets, ‘new’ for foils). Nine pairs of encoding/recognition epochs were employed. For each valence class, a total of nine pictures were presented during *both* encoding and recognition (Soloff et al., 2015). An event-related design was deployed to increase the flexibility of measurements by controlling for changes in baseline activation and to account for any confounding effects attributed to stimulus expectation or shifts in attention (Barron et al., 2016).

### Image and fMRI data analyses

All fMRI data were pre-processed using typical methods with Statistical Parametric Mapping 8 (SPM8). These methods included realignment to remove head movement artifact, normalization of the images into a spatially standard and ideal model, and smoothing to suppress noise or residual effects from differences in function. Low-frequency fluctuations were removed using a high-pass filter (1/256).

Responses to individual pictures were modeled using event-related models, with time and dispersion derivatives added to the model. Each participant contributed nine pairs of pictures to each valence (Negative, Positive). At the first level, contrast images (Encoding > Recognition) were used to quantify intra-subject effects of stimulus repetition for each valence class (Negative, Positive). Each participant thus contributed two contrast images each of which represented priming effects for Negative stimuli or priming effects for Positive stimuli. These images were carried forward to second-level random-effects analyses where Group (HC *v*. BPD) was modeled as an independent factor, Valence (Negative or Positive) was modeled as a non-independent factor, and participant age was modeled as a covariate. These analyses allowed for the age-controlled assessment of within-subject differences in activation changes (e.g. BPD _−ve_[_ENC>REC_]), and the subsequent assessment of between-group differences (BPD_−ve_[_ENC>REC_] ≠ HC_−ve_[_ENC>REC_]; BPD_+ve_[_ENC>REC_] ≠ HC_+ve_[_ENC>REC_]) in the effects of valence on priming. Significant clusters were identified using 10^4^ Monte Carlo probability simulations of the data (*p* < 0.05 cluster level) within each region of interest of an identified peak (Maldjian, Laurienti, Kraft, & Burdette, 2003) to compute the probability of a random field of noise (after spatial correlations of voxels based on image smoothness are accounted for). These simulations generate a minimum cluster size threshold for significance within a region of interest after thresholding noise to a certain level (Morris et al., 2018; Muzik, Baajour, Bressler, & Diwadkar, 2020); therefore, the analyses used *both* height thresholding (*p* < 0.05) and a minimum cluster size thresholding in arriving at a statistically robust threshold for identifying inter-group differences. The statistical approach is based on an underlying and tenable assumption that activation occurs over contiguous voxels whereas noise does not aggregate in clusters (Ward, 2000).

## Results

### Subject characteristics

The sample included 25 healthy control and 40 BPD participants (see online Supplementary Table S1 for demographic information).

### Episodic memory task fMRI results

**Fig. 1.**
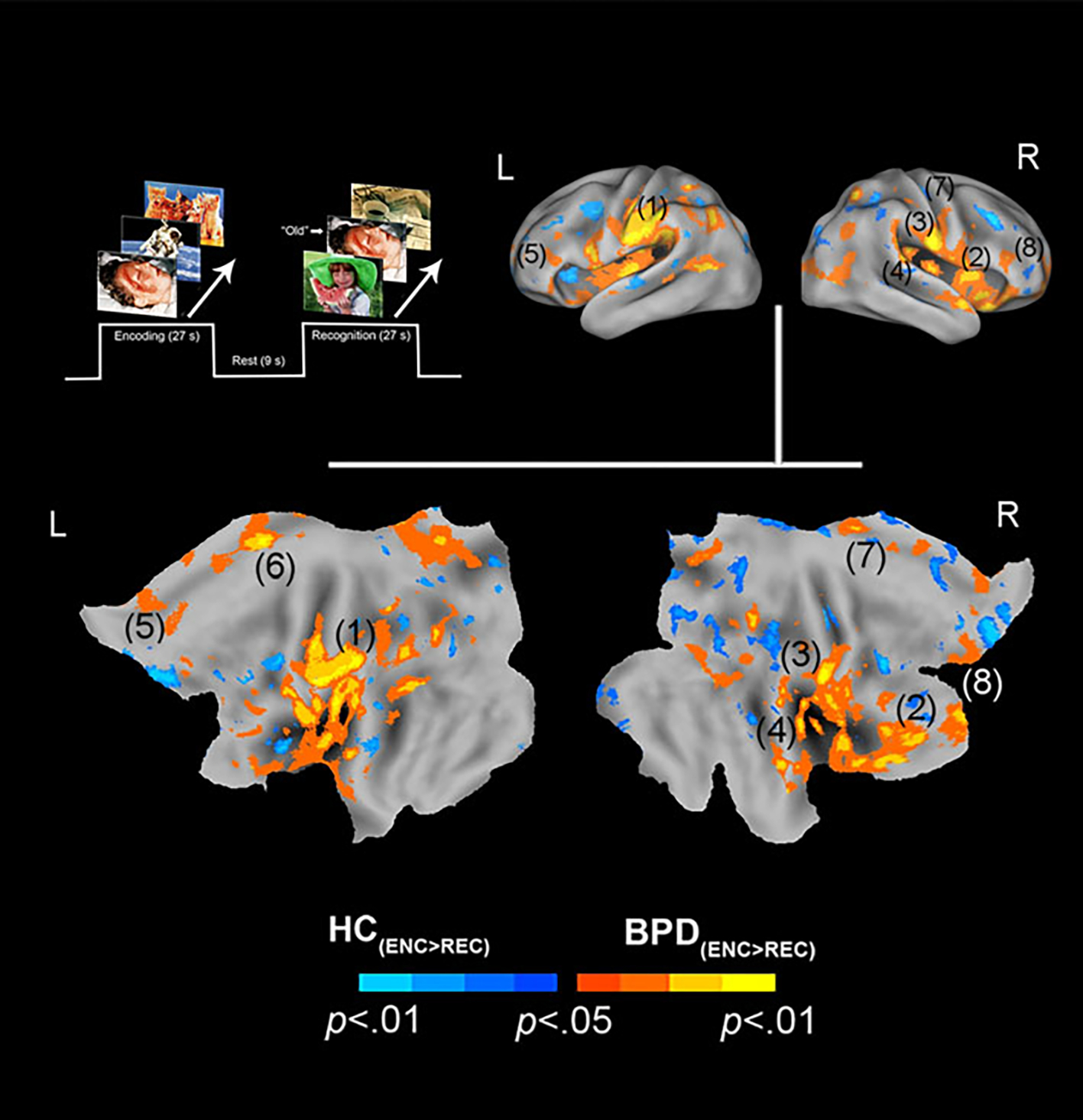
Negative valence and priming. The paradigm and conditions are schematically depicted (upper left). Inter-group differences in priming for negatively valenced pictures are overlaid on bilateral cortical surfaces, which are then unfolded onto a flat map for comprehensive rendition. As shown in the legend (bottom), warm colors reflect greater priming in BPD (BPD_−ve[ENC>REC]_ > HC_−ve[ENC>REC]_), while cool colors reflect the converse (HC_−ve[ENC>REC]_ > BPD_−ve[ENC>REC]_). To emphasize, warm colors depict greater priming for negatively valenced pictures in BPD participants. The significance peaks are denoted in (parenthesis). The numbers in the parenthesis are indexed in Table 1.

**Fig. 2.**
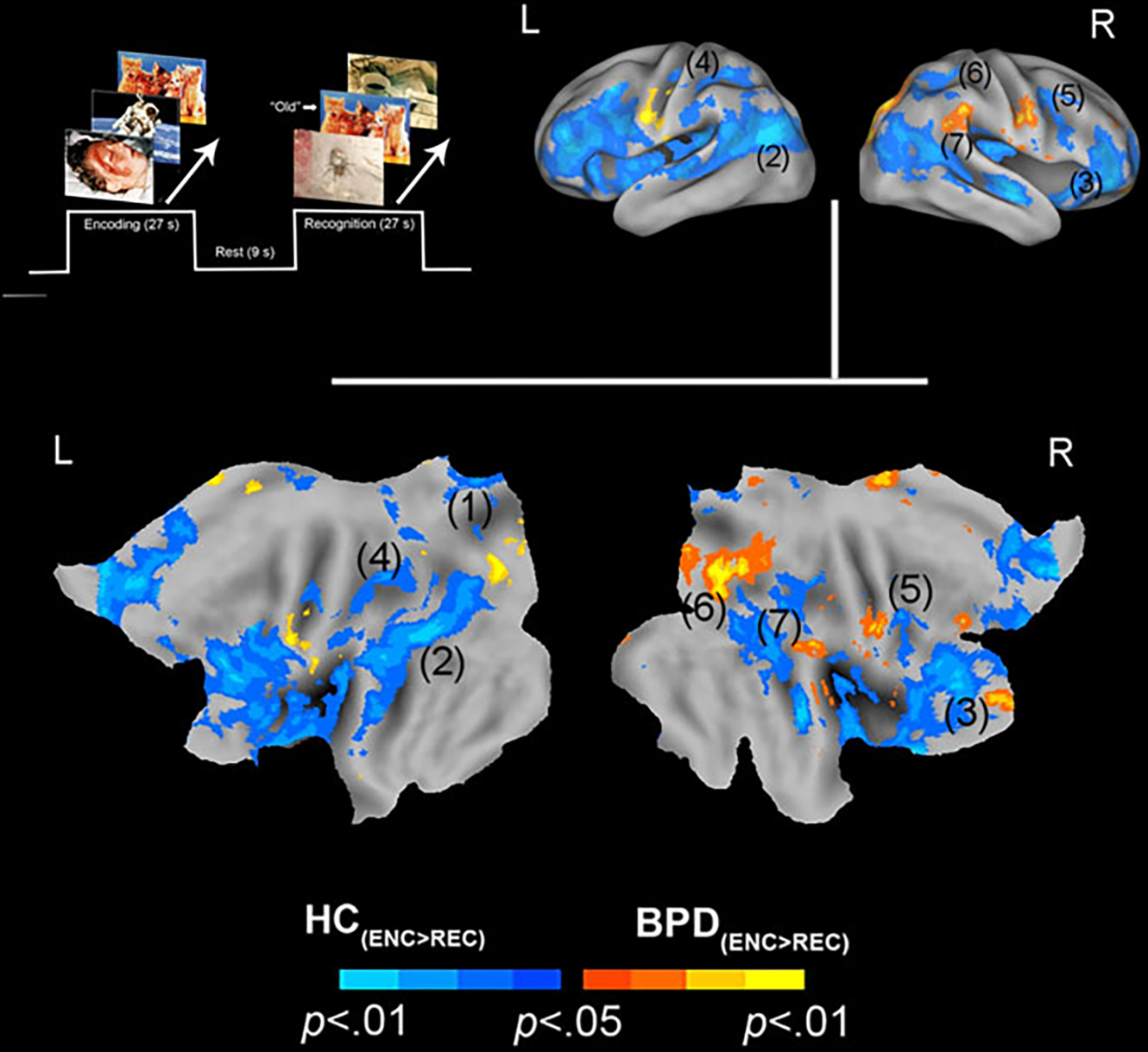
Positive valence and priming. The paradigm and conditions are schematically depicted (upper left). Inter-group differences in priming for positively valenced pictures are overlaid on bilateral cortical surfaces, which are then unfolded onto a flat map for comprehensive rendition. As shown in the legend (bottom), warm colors reflect greater priming in BPD (BPD_+ve[ENC>REC]_ > HC_+ve[ENC>REC]_), while cool colors reflect the converse (HC_+ve[ENC>REC]_ > BPD_+ve[ENC>REC]_). To emphasize, cool colors depict greater priming for positively valenced pictures in HC participants. The significance peaks are denoted in (parenthesis). The numbers in the parenthesis are indexed in Table 1.

**Fig. 3.**
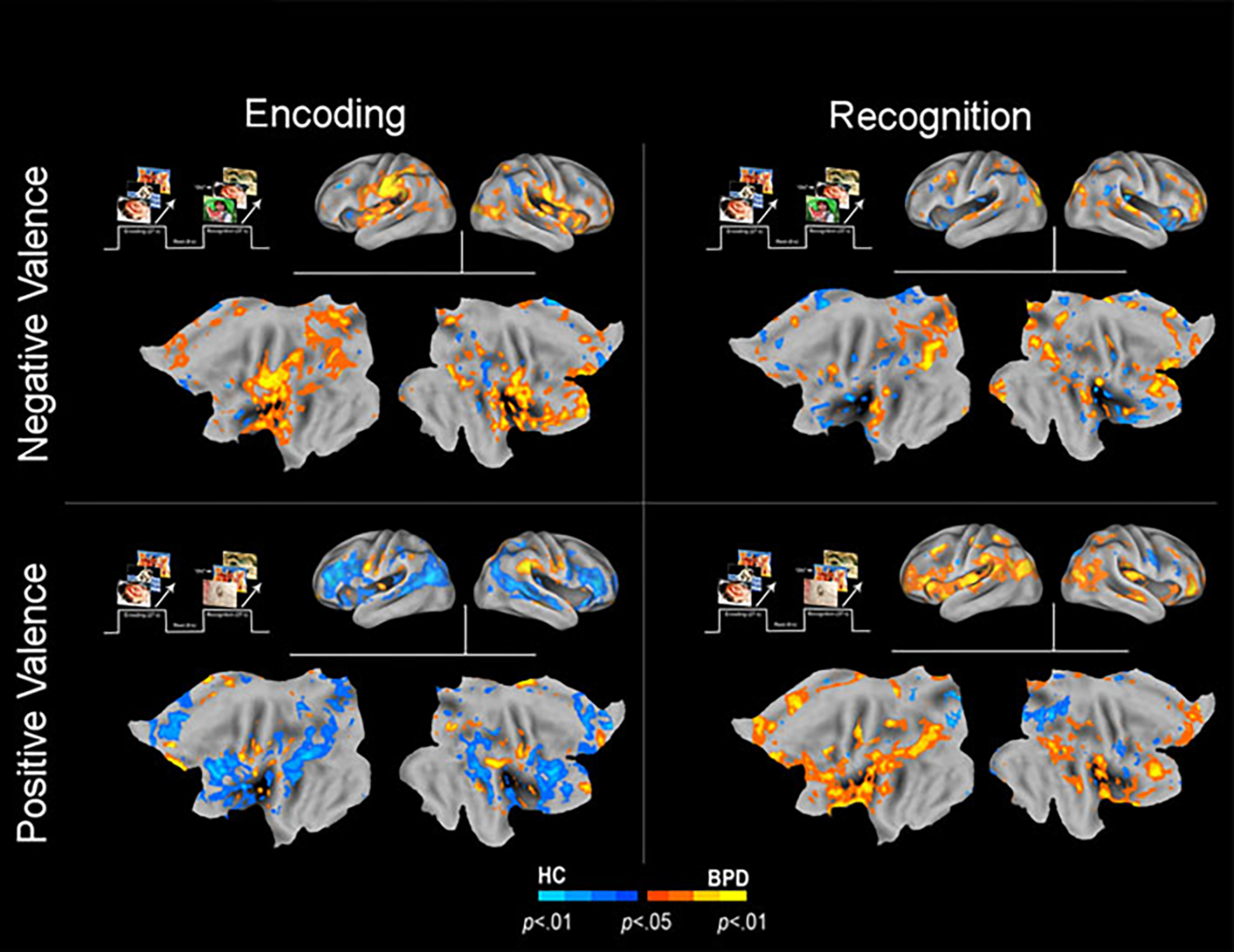
Inter-group differences are depicted in an overall condition (Encoding *v*. Recognition) × Valence (Negative *v*. Positive) framework, to provide a comprehensive overview of the observed effects. As with the previous convention, warm colors depict increases in BPD, while cool colors depict increases in HC. The upper left quadrant shows significantly greater activation among BPD subjects for *negatively* valenced stimuli compared to HC. The lower left quadrant shows significantly greater activation among HC subjects for *positively* valenced stimuli compared to BPD. The upper right quadrant shows significantly greater activation among HC subjects for *negatively* valenced stimuli compared to BPD. The lower right quadrant shows significantly greater activation among BPD subjects for *positively* valenced stimuli compared to HC.

**Fig. 4.**
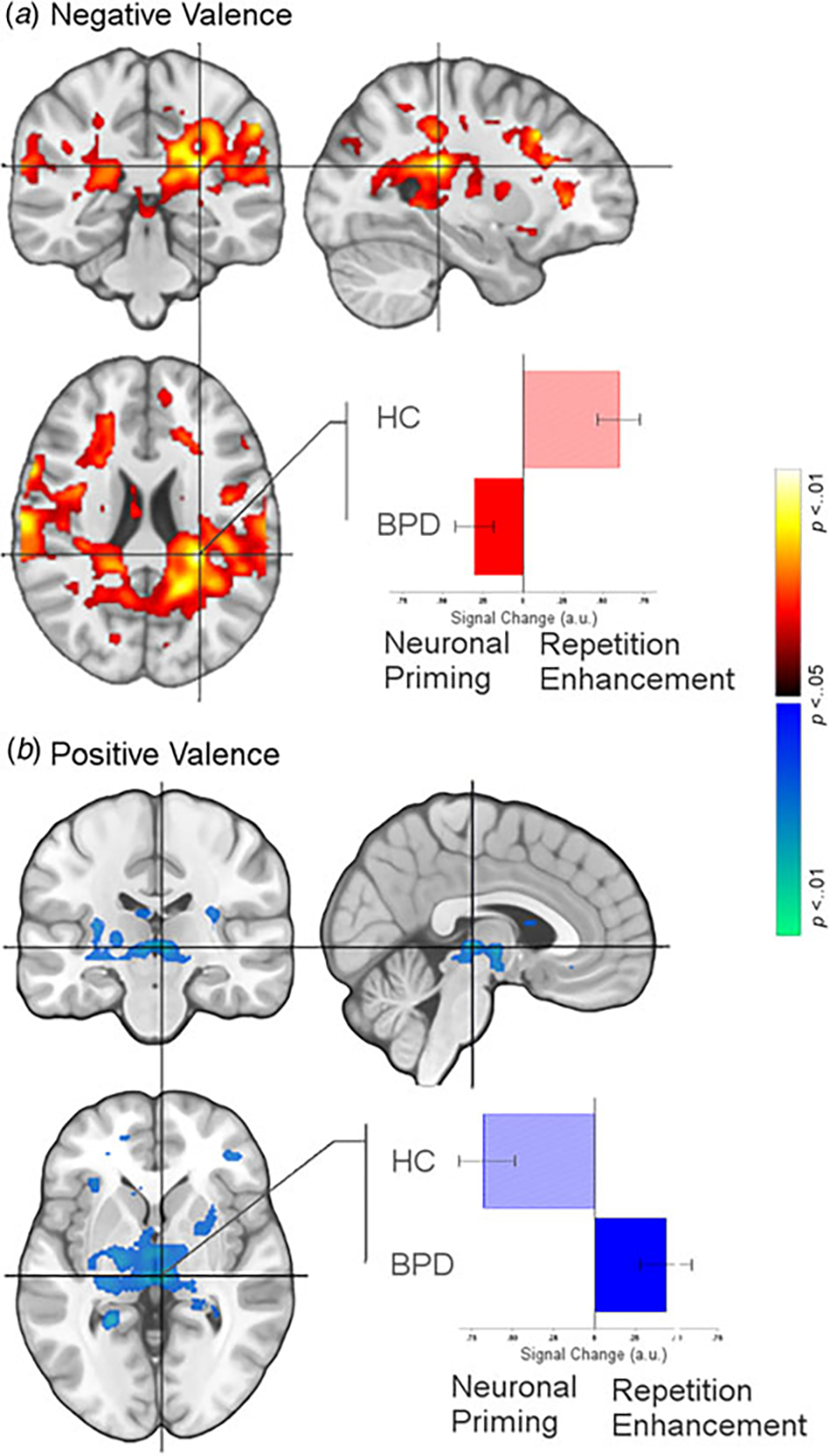
(*a*) Effects related to priming (BPD) but enhancement (HC) for negatively valenced stimuli are depicted under the peak in the inferior parietal cortex (see Table 1). The bar graph reflects the mean signal change from Encoding to Recognition for each group under the peak (extracted from sphere, 4 mm radius, error bars are ±s.e.m.). As denoted, leftward bars denote the effects of repetition priming under the peak, whereas rightward bars denote the effects of repetition enhancement. (*b*) The converse effects are depicted for positively valenced stimuli, with the graph elucidating changes under the peak in the thalamus. Figure 4 supplements Fig. 3, elucidating the nature of our observed effects as a function of valence.

We organize the presentation of results as follows: (1) We first present results of between-group analyses associated with negative valence (BPD_−ve_[_ENC>REC_] ≠ HC_−ve_[_ENC>REC_]) (Fig. 1), followed by (2) similar analyses for positive valence (BPD_+ve_[_ENC>REC_] ≠ HC_+ve_[_ENC>REC_]) (Fig. 2). (3) These analyses were extended (Fig. 3) to explore potential sources of the effects under (1) and (2). (4) Finally, for selected cluster peaks (Fig. 4), a graphical representation of the directionality of the effect provides a depiction of complementary patterns of priming or enhancement for each group (BPD, HC) under each valence (Negative, Positive).

In assessing inter-group differences for negative valence (BPD_−ve_[_ENC>REC_] ≠ HC_−ve_[_ENC>REC_]), multiple significant clusters (see Methods) were observed under the BPD_−ve_[_ENC>REC_] > HC_−ve_[_ENC>REC_] contrast. These were observed in frontal, cingulate, and motor regions including the pars triangularis, cingulum, pre-frontal cortex, and supplemental motor area. This result indicates that in BPD, the *decrease* in fMRI responses from the first to the second presentation of the negative stimuli was *greater* than that observed in HC, suggesting a sharper attenuation of the response between successive presentations of *negative* stimuli in BPD participants. The effects are depicted in Fig. 1 with statistical reporting of the peaks and cluster extents in Table 1. Peak localization in Table 1 was based on deterministic masks in stereotactic space (Maldjian et al., 2003), and the cluster extent reflects the size of the cluster around each observed peak.

In assessing inter-group differences for positive valence (BPD_+ve_[_ENC>REC_] ≠ HC_+ve_[_ENC>REC_]), we observed multiple clusters under the BPD_+ve_[_ENC>REC_] < HC_+ve_[_ENC>REC_] contrast. These were observed in multiple frontal and parietal regions, as well as the thalamus. This result implies that in BPD, the *decrease* in fMRI responses from the first to the second presentation of the positive stimuli was *less* than that observed in HC, suggesting a shallower attenuation of the response between successive presentations of positive stimuli in BPD participants. The effects are depicted in Fig. 2 with statistical reporting of the peaks and cluster extents in Table 1.

### Further elaboration of effects

Further analyses were designed to explore the potential source(s) of effects for both positive and negative valence. In these secondary analyses, we investigated if the effects under the clusters shown in Figs 1 and 2 were driven by differences in activation profiles during the initial presentation of stimuli (*Encoding*) (BPD[_ENC_] ≠ HC[_ENC_]) and/or the subsequent presentation of stimuli (*Recognition*) (BPD[_REC_] ≠ HC[_REC_]). To conduct these secondary analyses, we first constructed functional regions of interest (from Figs 1 and 2), used as spatial masks under which we assessed inter-group effects during Encoding and during Recognition.

Figure 3 (top row, left) shows that during the *initial* presentation (*Encoding*) of negatively valenced stimuli, BPD showed notably *greater* activation (BPD_−ve_[_ENC_] > HC_−ve_[_ENC_]), under the clusters in Fig. 1 (BPD_−ve_[_ENC>REC_] > HC_−ve_[_ENC>REC_]). This difference is less marked during the subsequent presentation (*Recognition*) though there is some evidence of reduced activation in key clusters. The elaboration of this effect suggests that the greater priming for negative valence in BPD was in part driven by greater activation during the initial presentation that may be related to the increased salience of negative valence in this group (see Introduction).

By comparison, during the initial presentation of positively valenced stimuli (bottom row, left), BPD showed notably lower activation (BPD_+ve_[_ENC_] < HC_+ve_[_ENC_]), under the clusters in Fig. 2 (BPD_+ve_[_ENC>REC_] < HC_+ve_[_ENC>REC_]). This effect is the converse of that observed under negative valence, and is consistent with the notion that positively valenced stimuli have increased salience in the healthy brain. The effects during the subsequent presentation (bottom row, right) suggest a notable increase in BPD participants, consistent with a notion of enhancement under repetition.

To better articulate these dissociated effects relating to valence, Fig. 4 (a subset of Figs 1 and 2) depicts the dissociated effects of priming and enhancement under the same cluster for negative valence (a) and positive valence (b), but with adjoining graphs that elucidate the nature of the effect for each. The crosshairs across each orthoview are centered on the significance peak for the inferior parietal cortex (a) and the thalamus (b) (see Table 1). The bar graphs reflect the mean signal change from Encoding to Recognition for each group under the peak (extracted from sphere, 4 mm radius). As denoted, leftward bars denote the effects of repetition priming under the peak, whereas rightward bars denote the effects of repetition enhancement.

## Discussion

In the context of an episodic memory task with stimuli of varying valence, we investigated the effects of valence on neuronal priming in BPD patients compared to healthy controls. Our salient results were: (1) BPD subjects displayed neuronal *priming* for *negatively* valenced stimuli (Fig. 1). This effect was driven by greater activation to negatively valenced stimuli during encoding (the initial presentation) compared to recognition (the second presentation) (Fig. 3, top row); (2) conversely, BPD patients evinced repetition *enhancement* for *positively* valenced stimuli (Fig. 2). This effect was driven by greater activation to positively valenced stimuli during recognition rather than encoding (Fig. 3, bottom row).

We forward a plausible explanation of our results: (a) the heightened salience of negatively valenced stimuli (which leads to greater activation during Encoding) evokes a state of affective familiarity in BPD. Evidence for affective familiarity has been documented in the literature in studies with healthy controls (Garcia-Marques et al., 2016); (b) this familiarity in turn drives the effect of neuronal priming for negatively valenced stimuli in this clinical group (Fiebach et al., 2005); (c) by comparison, reduced salience for positively valenced stimuli (which leads to greater activation in HC during Encoding) evokes a state of affective novelty in BPD, which in turn drives the effect of repetition enhancement in this clinical group (Subramaniam et al., 2012). In the remainder of the Discussion, we motivate the basis for these interpretations (based on extant studies) and highlight the clinical significance of our findings. We first place our results in the context of previous imaging studies in repetition priming/enhancement, and relate the specific brain regions that the effects were observed in, with some of the extant literature. Next, we sample evidence from (both functional and structural) imaging studies in BPD to provide a clinical framework for our effects. We conclude with a section on the clinical relevance of our results, and some limitations that constrain the interpretation and generalizability of the findings.

### Comparison to previous imaging studies

#### Normative studies of brain function

In experimental contexts, both positive and negative valence hold substantial salience in the typical human brain and underpin brain networks that drive social interactions (Nummenmaa et al., 2012). However, whereas positively valenced stimuli are ‘preferred’ in healthy controls (Nielen et al., 2009; Winecoff et al., 2013), negatively valenced stimuli are more salient to patients with BPD (Soloff et al., 2015; Soloff, Chowdury, & Diwadkar, 2019). Positive valence carries higher emotional salience presumably because the presentation of stimuli with positive valence activates latent representations of existing connections associated with positive life experiences (Lempert, Speer, Delgado, & Phelps, 2017; Pool, Brosch, Delplanque, & Sander, 2016; Reggev, Bein, & Maril, 2016). Indeed, meta-analyses of over 200 unique studies, which have employed a combination of valences with attention tasks, have suggested that this salience reflects an attentional bias in favor of positive over negative or neutral stimuli (Pool et al., 2016). By inference, such a bias reflects and/or results in a pattern of affective familiarity with this stimulus class (Garcia-Marques et al., 2016). Indeed, the directionality of our effects [Priming_(HC, +ve valence)_ > Priming_(BPD, +ve valence)_; Priming_(BPD, −ve valence)_ > Priming_(HC, -ve valence)_] motivates similar explanations, but only distinguished by the role of stimulus valence for each group.

Familiarity (and novelty) shows demonstrable representations in fMRI-measured brain activity. For example, *familiar* stimuli are more efficiently recalled, suggested by the fact that during their retrieval/recognition, evoked responses are reduced in multiple regions including frontal, parietal, and temporal cortices (Gilmore, Kalinowski, Milleville, Gotts, & Martin, 2019; Reggev et al., 2016). This change in responses implies not only memory, but also *shifts* in attention away from familiar stimuli may be at play (Thakral, Jacobs, & Slotnick, 2017). Recent work on attention shifts has shown that the BOLD response in areas like the parietal cortex changes when target features in the stimulus (e.g. stimulus color or stimulus location) are repeated (Brinkhuis, Kristjansson, Harvey, & Brascamp, 2020), suggesting that the neuronal signatures of priming are related to attention shifts *away* from repeated stimuli. In our study, the ‘feature’ that repeated was the stimulus identity itself, but the interactive role of attention with memory mechanisms is a known property of memorial processing. During memory processing, attention appears to exert substantial network effects (Ray et al., 2020), and appears to amplify priming effects in the parietal cortex. Effects related to attentional shifts may also explain the observed peaks in the thalamus, the principal sensorial gateway to the cortex (Barron, Eickhoff, Clos, & Fox, 2015; Saalmann & Kastner, 2011). Indeed, response properties of the thalamus are susceptible to the effects of attentional control (Jagtap & Diwadkar, 2016), and the evidence of priming in this structure suggests links with attention or pre-attention. Thus, the effects observed in healthy controls are consistent with extant knowledge on the representation of positive valence in the healthy brain. The converse of these effects was observed for negative valence in BPD patients (Fig. 1), largely in the same regions with additional differences observed in the mid cingulate cortex and the supplementary motor area. There is precedence for these effects in prior work in which priming and familiarity are associated with common activity in both the superior parietal and motor cortices (Thakral, Kensinger, & Slotnick, 2016), where the latter has been associated with facilitation in motor processing that may lead to reduced activity during familiarity and priming (Soldan, Zarahn, Hilton, & Stern, 2008). This rationale is likely to apply to the mid cingulate effects as well, given that this region occupies cortical space adjacent to the supplementary motor area, and is associated with motor control (Hoffstaedter et al., 2014). Regardless of the specific mechanisms, all the observed inter-group effects related to priming (Figs 1, 2, and 4 and Table 1) were observed in regions previously associated with priming, attention shifts, and memory (regardless of stimulus valence) (Dahlgren, Ferris, & Hamann, 2020; Simons & Spiers, 2003). Thus, the plausible familiarity/salience of positively valenced stimuli induces efficient recall of the memory trace in healthy controls. In effect, this places our interpretation of our results firmly in the domain of extant research that employs priming to identify the forward effects of memorial representations either using behavior or neuroimaging (Buckner et al., 1998; McNamara & Diwadkar, 1996).

#### Borderline personality disorder studies

BPD has diverse origins but the resultant long-term effect of the diagnosis is the disruption of mechanisms of emotion regulation particularly in the context of *negatively* valenced stimuli (Soloff et al., 2015). In terms of a mechanism, dysregulation may be coupled with/result from a heightened salience for negative stimuli, expressed through excessive rumination of, and disproportionate (and presumably more efficient) access to, negative memories (Baer, Peters, Eisenlohr-Moul, Geiger, & Sauer, 2012). Indeed, BPD subjects have an attentional bias toward negative stimuli, specifically faces expressing fear, and show a faster response time to congruent fearful face pairs and a slower response time to incongruent pairs or other emotions (Jovev et al., 2012). They also experience a stronger cognitive appraisal of emotion when presented with negative affective stimuli (Levine, Marziali, & Hood, 1997).

Behavioral habituation is a well-established phenomenon in healthy controls, with several studies examining habituation-related impairments in BPD patients (Hazlett et al., 2012). The notion of habituation is strongly related to emotional/behavioral processes, but is underpinned in variable ways by neuronal priming. Thus, Koenigsberg et al. (2014) observed a lack of habituation to the repeated presentation of negatively valenced pictures in BPD patients. Whereas controls showed a significant *increase* in activation in areas such as the mid cingulate, BPD patients showed a non-significant decrease in the reduction of the fMRI response in the same region (Koenigsberg et al., 2014, Fig. 1). From our perspective, their results showed greater ‘neuronal priming’ in BPD (though their effects did not reach significance, whereas our effects did). Notably, these (and other) studies have addressed subjective distress in association with the neuronal responses to negative stimuli, though as noted earlier, we relied on implicit stimulus-driven changes in neuronal activation to the presentation of repeated stimuli in the context of episodic memory.

An increase in the cognitive appraisal of negative stimuli in addition to heightened salience could contribute to the dysregulation in the effective processing of negatively valenced stimuli that BPD subjects experience. Studies have found that BPD subjects exhibit greater affective instability over healthy controls, marked by the lack of an increase in activation upon repeated presentation of negative stimuli (Denny et al., 2018; Koenigsberg et al., 2014). Thus, negatively valenced stimuli appear more likely to evoke negative representations that are inherently more familiar to patients, and may impede emotional regulation, an interpretation consistent with the studies of Koenigsberg et al. (2014). Consistent with this speculation is evidence that BPD participants show more neuronal priming for negatively valenced stimuli (though less behavioral habituation). Thus, our results are broadly consistent with the evidence of the heightened salience of negative stimuli in BPD subjects (Baer et al., 2012).

A converse of the ‘negative bias’ is an anti-bias to positively valenced stimuli, because the same life experiences that heighten the salience for negative stimuli also diminish the salience of positive stimuli. Studies have shown that BPD subjects evaluate positive experiences and situations as being less positive (Reichenberger et al., 2017). For instance, when watching a video with an actor saying a positive statement, BPD subjects describe their emotions to be higher on scales of anxiety, guilt, or embarrassment and lower on scales of happiness or pride compared to healthy controls (Reichenberger et al., 2017). This has been attributed to an inability to recognize those emotions in situations that are not as frequently experienced by patients. BPD subjects would therefore be expected to display an increase in activation following the repeated presentation of (relatively unfamiliar) positive stimuli. Indeed, this repetition enhancement was noted in BPD subjects for positively valenced stimuli. Although there is no study directly investigating the effects of repetition enhancement in BPD subjects, the evidence to support the basis for stimulus novelty as a mechanism for repetition enhancement in healthy control subjects is abundant, as is the evidence that BPD subjects are more familiar with/have a bias toward negative stimuli and are therefore less familiar with positive ones.

### Clinical relevance

Our results are clinically relevant to the study of BPD subjects, not just for memory processing and recall, but also with respect to differences in processing and recall associated with valence. These results provide us with some insight into the inner functioning of the BPD brain and potentially how regional and network differences between BPD and healthy brain function may stem from environmental (life experiences) as well as structural (neuronal processing) differences. Further investigation into the mechanisms underlying these results could provide a valuable understanding of BPD and its effects.

### Limitations

#### Extraneous and confounding effects

As mentioned previously, BPD cannot be randomly assigned to subjects, increasing the effects of extraneous variables on the results. BPD subjects may react or respond to the task differently than HC subjects due to the characteristics of the disorder itself. The use of clinical control groups could be added to future studies to help account for any effects contributing to BPD subjects’ results. It would likely decrease the limitation, but not fully eliminate it. Additionally, BPD subjects were far more likely to be victims of abuse in comparison to the healthy control subjects (60.0% and 0.0%, respectively). This could lead to a confounding relationship between the effects seen for negatively valenced stimuli, as well as differences in BPD responses between those that experienced abuse and those that did not (de Aquino Ferreira et al., 2018), though we point out that this abuse is itself a cardinal driver for the emergence of the disorder. Finally, our study was not designed to distinguish the explicit effects of the task (i.e. the task itself was an explicit episodic memory task) from the inferred implicit effects of valence (i.e. what implicit effect the stimulus valence exerted on emotional responses, and therefore priming). Disambiguating these issues would demand extensive psychophysical and behavioral studies.

#### Psychoactive medication

Forty-five percent of the BPD subjects were taking psychotropic medications at the time of the scan (*n* = 18). To investigate if medicated status exerted an influence on fMRI measures, two-sample *t* tests were performed to compare parameter estimates between BPD sub-groups (medicated *v*. non-medicated) at significant peaks (Table 1). No significant differences (*p*’s = 0.31–0.43, Cohen’s *d* ≤ 0.137 on all tests) were observed between BPD subgroups. We submit that while participants were currently symptomatic, any differences between medication sub-types would be too small (and our current sample underpowered) to be identified in our analysis.

#### Clinical ‘specificity’

A significant percentage of our participants (>80%) with BPD also had a history of, or current major depressive and/or substance use disorder. Having a history of MDD is characteristic of participants with BPD, and past and recent magisterial surveys of the literature have directly addressed the conceptual bases of such co-morbidity (Koenigsberg et al., 1999; Winter, Elzinga, & Schmahl, 2014). Koenigsberg et al. have suggested that BPD and MDD co-occur because both phenotypes share biological features. Subsequent meta-analyses provide some support for the functional bases of these hypotheses, particularly during the processing of negative affective valence, where BPD and MDD patients show similarly altered activations in cingulate, frontal, and occipital cortices (Schulze, Schulze, Renneberg, Schmahl, & Niedtfeld, 2019). These findings have strengthened the notion of BPD being a disorder of ‘emotion dysregulation’ (Schulze, Schmahl, & Niedtfeld, 2016), and this dysregulation often leads to substance use as a maladaptive coping strategy (Snow, Balling, & Zimmerman, 2020). The issue of co-morbidity in BPD is challenging to tease out precisely because the ‘psychosocial sequelae’ of MDD and BPD can contribute to the development of the other disorders and the ordering of diagnosis onset can be challenging to assess (Gunderson et al., 2004). In our case, patients were recruited from a longitudinal BPD study (see Methods) wherepatients with a primary diagnosis of BPD were well-characterized. While the rates of MDD in such patients decline over time, the prevalence remains high (Shah & Zanarini, 2018), confirming notions of a possible inextricable relationship between the two phenotypes. It is impossible for us (given the characteristics of our sample’s co-morbidity) to assess the marginal additions (or interactions) of MDD and SUD to the primary BPD diagnosis, and the impact on our results.

In this vein, the examination of other dimensional contributions including suicidality and childhood trauma (particularly sexual abuse) will be warranted in future investigations. We have shown that the lethality of suicidal attempts is related to fMRI responses to negative valenced stimuli used during sustained attention tasks (Soloff et al., 2019), consistent with altered fMRI profiles during self-reflection on aversive memories (Silvers et al., 2016). Childhood trauma (and particularly sexual abuse) is a known risk factor for BPD, with impacts particularly on medial temporal lobe structures such as the amygdala and hippocampus (Soloff, Nutche, Goradia, & Diwadkar, 2008), and also on brain network profiles during social cognition tasks (Duque-Alarcon, Alcala-Lozano, Gonzalez-Olvera, Garza-Villarreal, & Pellicer, 2019). The impacts of these long-term risk factors on brain responses are a very understudied area of the field. Understanding their effects on reflexive measures likef priming may help further sharpen the clinical focus of this research.

## Conclusion

Our results elucidate how ‘obligatory’ neuronal responses related to repetition priming in BPD are shaped by stimulus valence, and can motivate a more robust understanding of the impact of pathology on neuronal architectures. Our future studies are motivated by an interest in the discovery of the *network* bases of the observed effects, and may illuminate the bases of ‘dysconnection’ (a dominant theme in disorders such as schizophrenia) in BPD.

## Supplementary Material

Supplementary material

## Figures and Tables

**Table 1.** Information regarding the activation levels for each region of interest (ROI)

Number on figure (ROI)	Cluster Ext.	*p* (unc)	Peak (MNI)
Negative (ENC > REC) (Fig. 1)			
1. Inf. parietal L	8068	0.000	(−27, −34, 22)
2. Pars triangularis	299	0.000	(62, 12, 19)
3. Inf. parietal R	2029	0.000	(68, −13, 22)
4. Precuneus	908	0.001	(21, −39, 16)
5. PFC L	335	0.002	(−22, 24, 27)
6. Cingulum Mid. L	359	0.003	(−12, −19, 46)
7. Sup. motor area R	454	0.005	(14, −27, 51)
8. Inf. frontal R	71	0.009	(58, 26, 10)
Positive (ENC > REC) (Fig. 2)			
1. Thalamus L	25 622	0.000	(−18, −15, −2)
2. Inf. parietal L	2913	0.000	(−44, −69, 16)
3. Inf. frontal R	1515	0.001	(38, 39, −0)
4. Sup. parietal L	650	0.003	(−21, −49, 52)
5. Frontal Sup. R	126	0.007	(16, 41, 28)
6. Sup. parietal R	156	0.008	(40, −51, 49)
7. Temporal Mid. R	1300	0.01	(51, −55, 15)

N/A, not applicable.

The number of the anatomical ROIs corresponds to the number used to label the previous Figs 1 and 2 for negatively and positively valenced stimuli. The cluster extent indicates the voxel size of the activation region. The voxel peak represents the coordinates of the denoted peak.

Online Supplementary Table S1. Demographic information of subjects. Because groups differed in age (*t*_59_ = 4.15, *p* < 0.01), age was added as a covariate in all analyses of fMRI data.
