# Supplementary material for: Stimulus valence, episodic memory, and the priming of brain activation profiles in borderline personality disorder
Source: Psychol Med. 2021 Apr 16;52(16):4177–87. doi: 10.1017/S0033291721001136 (PMC9275123; doi:10.1017/S0033291721001136)
Supplement: Supplementary file 1 [file S0033291721001136sup001.doc]

|  | Mean | Female | Male | Abuse | | | Co-Morbidity | | | Psychoactive |
| --- | --- | --- | --- | --- | --- | --- | --- | --- | --- | --- |
|  | Age (*s.d.* ) | Subjects | Subjects | Physical (PA) | Sexual (SA) | PA/SA | MDD | SUD | MDD/SUD | Medication |
| BPD | 31 (*7.5* ) | 34 | 6 | 17 | 19 | 24 | 27 | 6 | 3 | 18 |
| HC | 24 (*6.0* ) | 23 | 2 | 0 | 0 | 0 | N/A | N/A | N/A | N/A |
